# Determinants of unmet family planning needs among women of reproductive age between 15 and 49 years in Siaya County, Kenya

**DOI:** 10.11604/pamj.2025.51.61.47943

**Published:** 2025-07-02

**Authors:** Ruth Anyango Ameso, Eliphas Gitonga, Isaac Ogweno Owaka

**Affiliations:** 1 Department of Environmental and Occupational Health, Kenyatta University, Nairobi, Kenya,; 2 Department of Family Medicine, Community Health and Epidemiology, Kenyatta University, Nairobi, Kenya

**Keywords:** Unmet needs, family planning, reproductive age

## Abstract

**Introduction:**

unmet need for family planning is still a major public health issue, impacting maternal and child health outcomes. In Kenya, 14% of women desire to avoid or delay a pregnancy but are not using contraceptives. Unmet need differs across counties, with Siaya having a high unmet need at 21% despite the target to eliminate unmet need for family planning. This study sought to establish determinants of unmet family planning needs among Women of Reproductive Age (WRA) between 15 and 49 years in Siaya County, Kenya. Specifically, the study sought to address three specific objectives: to determine the level of unmet needs among WRA, socio-demographic characteristics of WRA, assess the level of knowledge on family planning and to determine attitudes towards family planning among WRA.

**Methods:**

the study presents findings from the baseline phase of a larger quasi-experimental study design. It utilized a mixed research design. The study adopted the World Health Organization's 30 by 30 2-stage cluster sampling method. The FANTA sample size formula was applied to arrive at 724 respondents. A total of 728 women of reproductive age participated in the study. The study included sexually active WRA, married women, or women in a companion. The study targeted over 67,023 women already in such unions and sexually active, which served as the sampling frame for the study. The current study, being a baseline study, results after the health education intervention will be presented in another study. Results were presented in tables and figures. Ethical guidelines and procedures upheld during the study included informed consent, voluntary participation of participants, confidentiality, data security measures, assent for the minors, and usage of research licenses and approval from the ethics from the school. Data analysis was done using IBM SPSS version 28.0. The statistical analysis was undertaken in two steps: bivariate analysis and multivariate analysis

**Results:**

a total of 728 women of reproductive age participated in the study. The majority (45.2%) of the women were aged 25 to 34 years. Results showed that most (64.0%) of the women demonstrated a high level of knowledge, scoring 80% or above. However, only 2.7% had a low level of knowledge, with aggregate scores below 50%. The majority (75.5%) of the women had a positive attitude, with 24.5% having a negative attitude. The prevalence of unmet need was 52.7%. The proportion of unmet need was significantly more (COR = 1.61; 95%CI = 1.19 - 2.19; p = 0.002) among women with a low or moderate level of knowledge on family planning compared to those women with a high level of knowledge. Women aged 15 to 24 years were 3.43 times more likely to have unmet need for family planning compared to those aged 35 to 49 years (COR = 3.43; 95%CI = 2.23 - 5.26; p <0.001). Women with a negative attitude towards family planning had a significantly higher unmet need for family planning (COR = 1.53; 95%CI = 1.09 - 2.16; p = 0.015) compared to those women with a positive attitude.

**Conclusion:**

the study concludes that social-demographic factors such as education, age, or economic activity significantly influenced the unmet needs for family planning. Knowledge significantly influenced the unmet needs for family planning, where WRA with lesser knowledge were more likely to experience unmet needs. Lastly, Attitudes such as perceived harm of using contraceptives and stigmatization from their use significantly increased the odds of unmet family planning needs.

## Introduction

Maternal health is an important tenet of the Sustainable Development Goals (SDGs), which exposes the need to improve healthcare provision among women. The focus on maternal health has led to an increase in the birth control to minimize the mortality rates as per the SDGs [1]. Despite the campaigns on maternal health, the unmet needs for the use of birth control are still high, which exposes mothers to risks such as unsafe abortions and a high number of sexually transmitted diseases. Several studies have expressed the growing gap of unmet needs, which exposes women to risks. About 1.9 million girls between 15 - 49, of whereby about 1.2 billion needed birth pills. About 270 million of these girls had unmet needs by 2019, with the gap expected to widen to about 10% by 2030 [2]. Globally, the unmet needs stood at about 250 million in 2022, while the women of reproductive age who did not use contraceptives in the same period were about 214 million [3]. These figures reveal that the unmet needs are still high, which pushes women to use the traditional means of birth control, while others choose to live with the consequences. Studies have also shown that about 155 million people in the world do not use contraceptives, posing risks to the mothers and the unborn children [4]. The low and middle-income countries have the greatest chunk of the unmet needs for contraceptives, with the number expected to continue growing. This number is the highest in sub-African, which currently stands at 23%. This high number leads to unwanted pregnancies and high mortality rates among women. DRC Congo has the highest number of unmet needs at 33%, followed by Uganda at 28% and Nigeria at 25% [2].

Unmet needs are a global issue and have affected both the developed and the developing nations. In the US, for instance, about 3.4% of women between 15 to 45 years have unmet needs due to health concerns. These health concerns are due to individual-level beliefs, attitudes, and different preferences on family controls. Besides, medical conditions such as chronic illness have impacted the demand for family planning needs in the US [5]. In the UK, a significant number of women have unmet family planning needs, with issues such as socioeconomic status, age, and relationships playing a huge role. Younger women, particularly those between 20-24 years, have the highest rates of unmet needs. Besides, these women want to delay or stop childbearing but are not using contraceptives, which leads to unmet needs [6]. In Germany, there is a high unmet need for registered refugees and asylum seekers. A study revealed that the unmet needs for family planning can be as high as 23% for refugees and asylum seekers. Most of this population relies on less effective methods, such as withdrawals [7].

While Africa has made some significant strides in addressing the unmet needs for family planning, there are still some challenges. Lack of knowledge about birth control is the major cause of the high number of women with unmet needs. Other factors, such as behavioral requirements and objections from the men in the communities, also play a significant role in the high number of unmet needs. Religious and cultural influences men´s behaviors and their perceptions on birth control, which in turn affects unmet needs [8]. Women in rural areas are more likely to experience unmet needs compared to those in urban areas. These high numbers of unmet needs may lead to several unintended consequences, such as pregnancies and abortions. These risks are more prevalent in the underdeveloped countries and may push women to engage in unsafe abortions and exposing women to risks. Besides, unmet needs lead to unplanned pregnancies, which affect the children because the parents may not be ready to have the children [9]. Some of the issues that lead to high cases of unmet needs, especially in rural areas, include misconceptions, husbands´ disapproval, and cultural issues. Misconceptions about family planning lead to rumors that paint the usage of contraceptives in a bad light [10]. Women may also have bad perceptions of the side effects, while others may face disapproval from their husbands. In most developing countries, women may not even know where to get family planning [11]. Consequently, it is important to educate women on the importance of family planning in overcoming the unmet needs for contraceptives.

The Kenyan government has taken huge strides in addressing the challenges of unmet needs for birth control. The government's efforts have borne fruit, with the usage of family planning increasing significantly. For instance, the country has witnessed a huge positive growth between 1992 and 2023, which can be attributed to the government´s efforts [8]. Despite these efforts, however, the unmet needs remain high in the country. Specifically, there is a huge disparity between the unmet needs in the different counties in Kenya. The number of unmet needs in the country is moderately high, given the huge decline in the fertility rates. For instance, Lawrence *et al*. [3] explain that the fertility rates declined by 3% in the last three decades (between 1989 and 2023), which is higher compared to 6.7% in the previous period [12].

Although there is a huge decline in unmet needs for the use of birth pills, the decline has not been proportional in all countries. Some regions witness higher decline rates than others, leading to concerns. The decline in the unmet needs in the country has also been visible among the socio-economic divides in the country. This decline in the unmet needs is currently at 14%, which is still high [11]. The high fertility rates have increased the population growth, which has in turn accelerated the existence of unmet needs for family planning. The increase in unmet needs in birth control has had a huge impact on maternal and child health, leading to rapid population growth. Rapid population growth due to the increase in unmet needs has been identified as a huge challenge in resource utilization, thus affecting growth and development [13]. The increase in unmet needs has also exposed women to health challenges, which may lead to death. These challenges include maternal mortality, unsafe abortions, and mental health challenges. Kenya has witnessed a decline in total fertility rates between 1989 and 2022, with the country witnessing a 3.3% decline in the period [11]. The country has also witnessed an increase in the consumption of contraceptives, witnessing a 57% increase [14]. However, this increase is not uniform, as some counties have had higher increases than others. Certain regions, especially in ASALs, have continued to struggle with unmet needs for family planning. Currently, about 76% of married women and 89% of sexually active women have shown the desire to use family planning drugs.

In Kenya, younger women have experienced a higher risk of mistimed pregnancies and unwanted pregnancies compared to their older counterparts. Younger women aged 14-24 years have a higher chance of a mistimed pregnancy at 30% compared to all women (15-49) years at 15%. This challenge has meant that about 103 out of 1000 births in Kenya are delivered by girls in the 15-19 years age bracket. Incidentally, accidental pregnancy has led to abortions, maternity deaths, and financial hardships. The contraceptive use is also low among sexually active single women in ASALs compared to other regions in the country. The main reasons for the low uptake of contraceptives include perceived side effects, cultural limitations, and religious beliefs [12]. Physical and lack of financial resources to access the family planning methods is another major challenge. The health facilities that offer family planning are not the same throughout the country; some regions have better facilities than others. Women in counties with low uptake of family planning have complained of frequent stockouts.

The challenge of unmet needs of family planning has continued to persist and has become a major issue of concern in public health. Siaya County is one of the several counties in the country that has a high unmet need for family planning compared to the national average [15]. The County has also registered a very high fertility rate that surpasses the national average. Siaya County has one of the lowest median ages at first births in the country, a situation that exacerbates the challenges of unmet needs for family planning. Despite the government's efforts and commitment to enhance the uptake of family planning services, the unmet needs have continued to persist in Siaya County. Resource constraints, social-cultural barriers, and healthcare access barriers have contributed to the glaring challenge of unmet needs for family planning [16]. While the use of community-based interventions has been efficient in addressing the challenges in other regions, it is imperative to evaluate the effectiveness and suitability of mitigating the unmet needs for birth control in Siaya County. This study sought to examine the determinants of family planning needs among WRA in Siaya County.

**Objectives:** 1) To determine the level of unmet need for family planning amongst women of reproductive age in Siaya; 2) to determine the socio-demographic characteristics of women of reproductive age in Siaya County; 3) to assess the level of knowledge on family planning amongst women of reproductive age in Siaya County; 4) to determine attitudes towards family planning amongst women of reproductive age in Siaya County.

## Methods

**Study design:** the study presents findings from the baseline phase of a larger quasi-experimental study evaluating the effectiveness of a community-based health education intervention on reducing unmet need for family planning among women of reproductive age in Siaya County. The design was appropriate because it facilitated the collection of baseline data prior to the intervention. this baseline information is critical for identifying what is currently happening before the intervention is implemented; hence, difference in difference analysis will be achieved. The study adopted a mixed research design approach incorporating both quantitative and qualitative techniques to boost inferential leverage, therefore reducing bias through triangulation of findings. These findings focus on the quantitative baseline data, which were collected prior to the intervention. The chosen design allows for the study of the cause-and-effect relationship between the variables [17].

**Study setting:** the study was conducted in Siaya County, which is among 47 counties in Kenya. The County shares boundaries with Vihiga, Kisumu, and Busia counties. The research took place within Siaya County, chosen purposefully due to its notably elevated unmet need for family planning in contrast to the national average. Siaya has a geographical area of roughly 2500 square kilometers and has 6 sub-counties, 30 wards, and up to 98 villages. The County had a population of 993,183 in 2019 and a projection of 1,040,616 in 2022, according to statistics by the population census. It is projected that the population of the County will be roughly 1,136,553 persons by 2027. The population of the County is predominantly young people ranging between 15 and 29 years, who are also actively of reproductive age. The population of the young is estimated to grow from 234,870 in 2019 to 257,874 in 2022, 283,563 in 2025, and 307,175 by 2027. Among the sub-counties in Siaya, Alego Usonga is the largest, covering up to 605.8km^2^, while Ugunja is the smallest with an area of about 97.2km^2^.

**Participants:** participants from the study were drawn from Siaya County and included women of reproductive age, are also sexually active, and are in committed relationships or are married. However, the study excluded women who were critically ill. Siaya County has the highest level of unmet family planning needs at 21%. Available data from the Ministry of Health put the figure at 67,023 women in unions who are sexually active. The participants were from different religions, different marital statuses, occupations, age groups, and had different numbers of children. Critically ill and mentally ill individuals were excluded from the study.

### Variables

**Dependent variable:** the dependent variable for the study was unmet need for family planning. The dependent variable was dichotomous and categorized as either “unmet need” or “met need” for family planning purposes. The variable was measured by a set of questions adapted from the Measure DHS filters for Unmet needs for family planning. The met need comprised the percentage of those desiring to postpone pregnancy and were actively on contraceptives, whereas unmet need was defined by the category that desired to postpone, did not want to have any more children but were not actively utilizing contraceptives or those who reported being pregnant at the time of the study but the pregnancy was either unwanted or mistimed.

### Independent variables

**Socio-demographic characteristics:** age, education levels, occupation, and number of children.

**Level of knowledge on family planning:** understanding of what Family planning is, what family planning methods participants know, benefits of family planning, and knowledge on where to get the contraceptives.

**Attitudes towards family planning:** attitudes towards perceived benefits, risks, and belief attitudes.

**Data sources:** data collection was done using a semi-structured questionnaire and interviews. A questionnaire was designed and uploaded onto Kobo Collect for ease of storage and management. In-person administration of the tool was done where the research assistant sat with the participants and asked the questions in both local and Swahili languages to encourage participation. The exercise took one month. The data collected sought demographic information in the first section, attitudes towards the use of family planning in the second section, knowledge levels on family planning, and unmet needs for family planning. Interviews also collected data from the health officers through face-to-face interviews. Notes were taken during the interviews to gain insights from the participants regarding the subject.

**Study design and sampling:** FANTA sample size determination by Robert Magnani was used to determine the sample size of 724 women of reproductive age for this baseline study. The study adopted the WHO 30 by 30 two-stage cluster sampling method to get the number of women of reproductive age. A systematic approach using probability proportional to size was used. Multi-stage sampling method where the first stage involved the selection of villages as primary sampling units (PSUs - cluster). The second stage involved sections of the households with women of reproductive age that were in the villages as the secondary sampling units (SSUs). To determine the actual households to select, stratification was done according to the wards and the households in those wards. A list of villages with an estimated number of households was sought from the County administration. To minimize selection bias, all villages were sorted after assigning a random number and then stratified by ward. Probability proportional to size (PPS) sampling was done at the first stage of the selection of villages. Based on the actual number of households per selected village, total number of households per selected ward, and total number of households in the entire Alego Usonga sub-County and Rarieda sub-County, the overall sampling weight.

**A within-community sampling method:** in the second stage of sampling, the objective was to select a household with a woman of reproductive age. With the help of community health promoters (CHPs) and community health assistants in charge of community units, the field teams listed all households in the village. A systematic sampling method was implemented, initiated with a random start position between 1 and the sampling interval. The sampling interval was determined by dividing the total number of households in each village by 12 (the number of interviews per village). Where the list was less than the number required, all the households were visited. The teams were guided by the same CHPs to locate the selected households. FANTA sample size formula was used to determine the sample size of respondents for this quantitative study [17]. The FANTA sampling method is as shown in the following formula:

$$n=D*\frac{{\left(Z_{1-\infty /2}+Z_{1-\beta }\right)}^{2}*\left(P_{1}\left(1-P_{1}\right)+P_{2}\left(1-P_{2}\right)\right)}{{\left(P_{2}-P_{1}\right)}^{2}}$$

P_1_ signifies the initial estimation of unmet need for family planning, standing at 27%. P_2_represents the final estimation of unmet need for family planning, recorded at 15%. The difference between P_2_ and P_1_ indicates the estimated change over time, which amounts to 12%. The symbol α denotes the type 1 error rate, set at 0.05. β denotes the Type 2 error rate, which is established at 0.20. Z_α/2_ signifies the standard normal deviate at the desired statistical significance level, calculated as 1.96. Z_β_ denotes the standard normal deviate at the desired statistical power, equating to 0.84. D stands for the design effect, determined to be 2. The total sample of households with women of reproductive age was 724 households/women in both intervention and control sites according to the FANTA formula.

**Statistical methods:** the analysis commenced with descriptive statistics for all factors: socio-demographic characteristics, knowledge on family planning, source of information for family planning, attitude towards family planning, and factors associated with unmet need of family planning. Results for each variable are presented as counts and percentages for categorical variables, and as measures of central tendency for continuous variables - mean (SD) for normally distributed; median (IQR) for skewed distribution. Bivariate analysis: to identify factors associated with unmet need of family planning (before adjustment for confounding), crude estimation of the statistical significance of the association between unmet need of family planning with each independent variable (knowledge on family planning, attitude towards family planning was conducted using Pearson Chi-Square test or Fisher exact test (informed by the mean expected counts per cell) for categorical variables. Crude odds ratios with corresponding 95% confidence intervals, calculated with test-based methods, were estimated to measure the strength of association with the unmet need for family planning. The threshold for statistical significance was set at p<0.05 (2-tailed) for all inferential statistics. Statistical analysis was performed using IBM SPSS version 29.0.

**Ethical considerations:** three bodies were critical in obtaining consent from participants: The National Commission for Science and Technology (NACOSTI) of reference number 404944, the Ethical Review Board of Kenyatta University of reference number PKU/2996/12020, and Siaya County Health Management. Further, to ensure confidentiality, the data was encrypted and protected using passwords to enhance the integrity of the data and prevent unauthorized access to the data. Names and personal details of the participants were not directly linked to the study. Participation in the study was voluntary without any imposed risks or direct financial benefits for the participants. For the WRA who participated in the study and were below 18 years, the assent was obtained from their spouses if they were above 18 years or someone in the family who was an adult.

## Results

**Figure 1:**
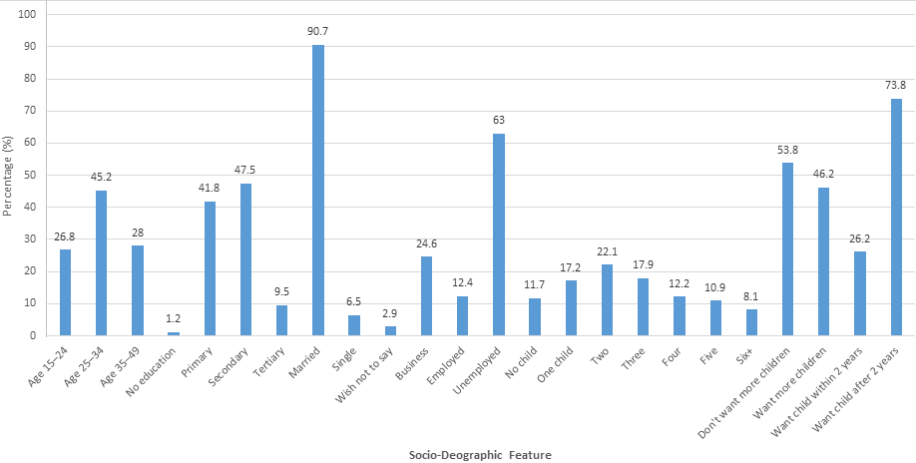
socio-demographic characteristics of women of reproductive age in Siaya County

**Socio-demographic characteristics among women of reproductive age 15 to 49:** socio-demographic characteristics among women of reproductive age in Siaya County are summarized in Figure 1. A total of 728 women of reproductive age participated in the study, with a response rate of 100.55% (728 out of 724). The highest percentage (45.2%) of the women were aged 25 to 34 years. Slightly lower than half (47.5%) had a secondary level of education, while only 9.5% attained a tertiary level of education. Most (90.7%) of the women were married, with the highest percentage (22.1%) having two children. The majority (63.0%) reported being employed. More than half (53.8%) expressed currently no desire for more children, and most (73.8%) preferred to have a child after at least two years.

**Descriptive statistics on knowledge of women of reproductive age on family planning:** knowledge regarding family planning was assessed as indicated in Table 1, which revealed that a large percentage (95.1%) had received information about family planning. Among those who had information, the majority (62.6%) recognized that family planning is used for controlling the number of children. Similarly, 97.4% of the women were aware of at least one family planning method, with implants (74.6%), injectables (73.1%), and pills (61.9%) being the most commonly recognized methods. However, lactational amenorrhea (5.6%) and male sterilization (6.6%) were among the least recognized family planning methods. Regarding benefit awareness, the majority (97.0%) of the women were aware of the advantage of using contraceptives, with most (71.0%) and (49.2%) acknowledging their benefit in spacing births and preventing unplanned pregnancies, respectively. Most (60.9%) expressed that they were very confident in their awareness of the different types of contraception available for family planning, while only 1.1% indicated that they were not confident at all.

**Table 1:** knowledge on family planning among women of reproductive age in Siaya County

Knowledge	Frequency (N=728)	Percent (%)
**Receipt of any information on family planning**		
No	36	4.9
Yes	692	95.1
Understanding of the term family planning*		
Controlling the number of children	456	62.6
Spacing between pregnancies	399	54.8
Preventing unintended pregnancies	286	39.3
Choosing when to start a family	153	21.0
**Awareness of any family planning methods**		
No	19	2.6
Yes	709	97.4
Types of family planning methods known*		
Male sterilization	47	6.6
Pills	439	61.9
intrauterine device (IUD)	211	29.8
Injectable	518	73.1
Implants	529	74.6
Male condom	370	52.2
Female condom	195	27.5
Emergency contraception	106	15.0
Rhythm withdrawal method	65	9.2
Lactational amenorrhea (LAM)	40	5.6
**Awareness of the benefits of using contraceptives**		
No	22	3.0
Yes	706	97.0
Benefits of family planning*		
Helps in spacing families	501	71.0
Improved maternal health	286	40.5
Economic stability	180	25.5
Reduction of unintended pregnancies	347	49.2
**Confidence in understanding the contraceptives for family planning**		
Not at all confident	8	1.1
Slightly confident	96	13.2
Moderately confident	143	19.6
Very confident	443	60.9
Extremely confident	38	5.2

**Figure 2:**
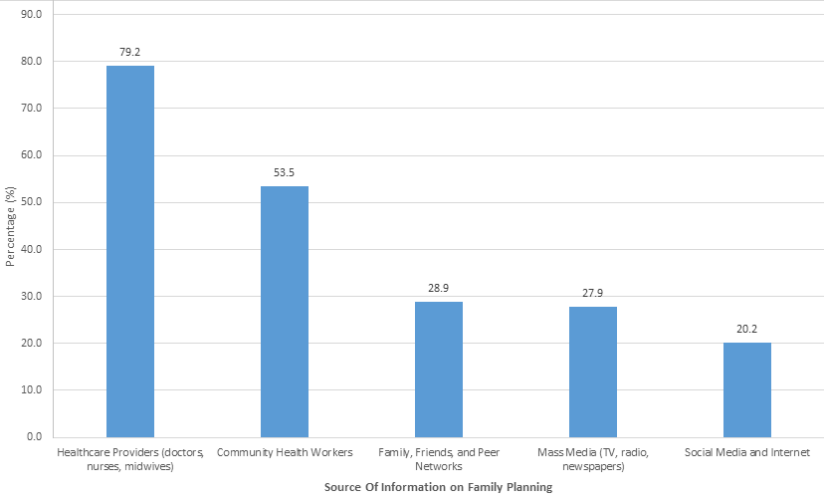
source of information on family planning for women of reproductive age in Siaya County

**Source of information for family planning:** results revealed that healthcare providers, including doctors, nurses, and midwives, were the main source of information about family planning, accounting for 79.2%. This was followed by community health workers, who contributed 53.5% as indicated in Figure 2.

**Figure 3:**
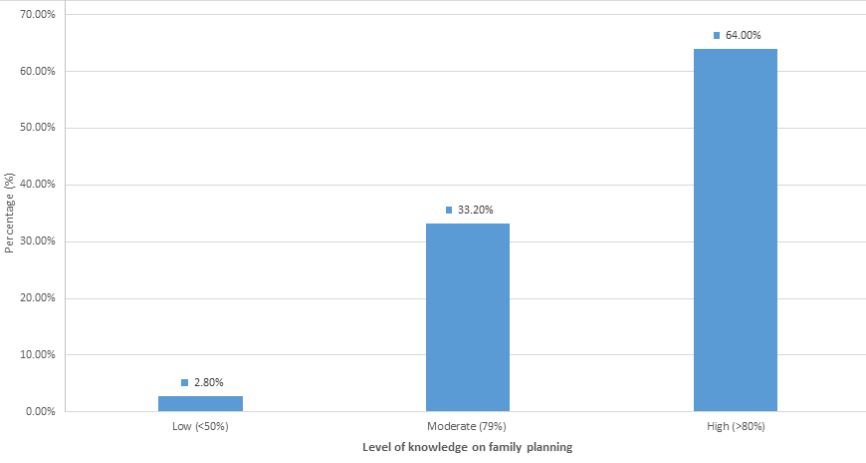
levels of knowledge on family planning among women of reproductive age in Siaya County

**Level of knowledge on family planning:** the level of knowledge on family planning was assessed based on the score assessment aggregation. The maximum score was 7, and the minimum score was 0. The average mean score was 4.54. The scores were converted into percentages. Those who scored above 80 were considered to have high knowledge. Most (64.0%) of the women demonstrated a high level of knowledge, scoring 80% or above. However, only 2.7% had a low level of knowledge, with aggregate scores below 50% as indicated in Figure 3.

**Descriptive statistics on attitude towards family planning:** most (68.8%) of the women agreed that the family is beneficial for both their well-being and health. Similarly, 68.4% agreed with access to different family planning methods for making decisions that are important for reproductive health, and 65.7% of them agreed on the importance of open discussion between partners for healthy relationships. However, most (60.6%) of the women agreed that support by society for family planning is more judgmental than being supportive. Regarding confidence in accessing and using family planning, as well as raising awareness of family planning in the community, the majority of women agreed with these statements, with 71.2% and 70.1% respectively, as indicated in Table 2.

**Table 2:** attitude towards family planning

Statements	Strongly disagree n (%)	Disagree n (%)	Neutral n (%)	Agree n (%)	Strongly agree n (%)
Family planning benefits the well-being and health of women	7 (1.0)	6 (0.8)	66 (9.1)	501 (68.8)	148 (20.3)
Access to a variety of family planning methods is important in enabling women to make decisions that are important for their reproductive health	3 (0.4)	3 (0.4)	64 (8.8)	498 (68.4)	160 (22.0)
An open discussion between partners is crucial in fostering healthy relationships between spouses	5 (0.7)	0 (0.0)	90 (12.4)	478 (65.7)	155 (21.3)
Support by society on family planning is more judgmental than supportive	11 (1.5)	70 (9.6)	122 (16.8)	441 (60.6)	84 (11.5)
I am quite confident in accessing and use of family planning when needed	5 (0.7)	13 (1.8)	79 (10.9)	518 (71.2)	113 (15.5)
I believe creating awareness and knowledge on family planning needs to be promoted in the community	1 (0.1)	1 (0.1)	37 (5.1)	510 (70.1)	179 (24.6)

**Figure 4:**
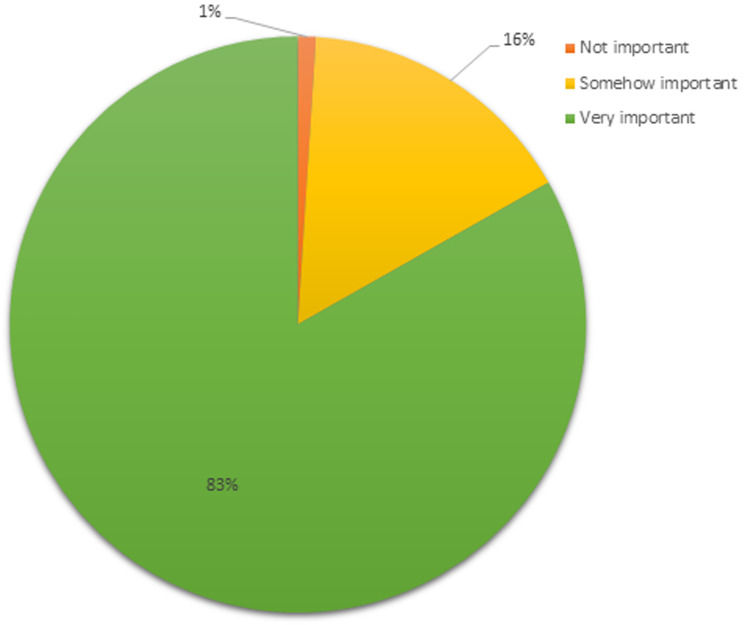
views on the importance of family planning for controlling the size of families among women of reproductive age in Siaya County

**Importance of family planning for controlling the size of families:** a majority (83%) of the women viewed that family planning is very important in controlling family sizes, 16% viewed it as somewhat important, while only 1.0% stated otherwise, as indicated in Figure 4.

**Descriptive statistics on unmet need of family planning:** about a quarter (28.6%) of the women wanted to avoid pregnancy for at least two years, and they were not using family planning methods. The main reason for not using any family planning methods was religion or culture (58.9%), followed by some health concern (18.4%). The majority (64.8%) of the women were currently using contraceptives, and among them, the main methods used were implants (45.8%) and injectables (33.3%). Although about one-fifth (22.4%) wanted to wait at least two years before having another child, they were not currently using any contraceptive. Those women who decided to have no more children, but were not currently using any method of contraception, were 16.1%. Among the women who were pregnant at the time of data collection (10.2%), 31.1% reported that their pregnancy was unplanned (Table 3).

**Table 3:** unmet needs of family planning among women of reproductive age in Siaya County

Variables	Frequency (N=728)	Percent (%)
**Ever wanted to delay or avoid becoming pregnant for at least two years, but you were not using any family planning methods**		
No	520	71.4
Yes	208	28.6
**Currently using any form of contraception to prevent pregnancy**		
No	256	35.2
Yes	472	64.8
**Would you like to wait at least two years before having another child, but are not currently using any contraception?**		
No	565	77.6
Yes	163	22.4
**Unmet need for Limiting- Have you decided not to have any more children, but are currently not using any method to avoid pregnancy**		
No	611	83.9
Yes	117	16.1
**Whether currently pregnant**		
No	654	89.8
Yes	74	10.2
**Whether the current pregnancy was planned**		
No	23	31.1
Yes	51	68.9

**Bivariate analysis for factors associated with unmet need for family planning:** bivariate analysis included testing the association between socio-demographic factors, level of knowledge, attitudes, and unmet family planning needs.

**Association between socio-demographic characteristics and unmet need for family planning:** a bivariate analysis of the relationship between socio-demographic characteristics and unmet need for family planning is shown in Table 4. Women aged 15 to 24 years had a significantly higher proportion of unmet need for family planning compared to those aged 35 to 49 years, with a crude odds ratio (COR) of 3.01 (95% Confidence Interval (CI) = 1.99 - 4.55; p <0.001). The proportion of women without formal secondary education had significantly more unmet need for family planning than those women with tertiary education (COR = 1.70; 95%CI = 1.01 - 2.86; p = 0.046). Single women were significantly (COR = 3.18; 95%CI = 1.59 - 6.38; p = 0.001) more likely to face unmet need for family planning compared to married women. Women with no children had a significantly (COR = 2.56; 95%CI = 1.25 - 5.25) higher proportion of unmet need for family planning compared with those women with six or more children.

**Table 4:** log odds on the association between socio-demographic characteristics and unmet need for family planning among women

Variables	Unmet need		Met need		COR	p value
	n	%	n	%		
**Age (years)**						
15-24	138	70.8	57	29.2	3.01	<0.001
25-34	155	47.1	174	52.9	1.11	0.573
35-49	91	44.6	113	55.4	Ref	
**Level of education**						
No formal education	7	77.8	2	22.2	4.29	0.082
Primary	145	47.7	159	52.3	1.12	0.677
Secondary	201	58.1	145	41.9	1.7	0.046
Tertiary	31	44.9	38	55.1	Ref	
**Marital status**						
Married	335	50.8	325	49.2	Ref	
Single	36	76.6	11	23.4	3.18	0.001
Wish not to say	13	61.9	8	38.1	1.58	0.318
**Occupation**						
Business	87	48.6	92	51.4	0.79	0.183
Employed	47	52.2	43	47.8	0.91	0.696
Unemployed	250	54.5	209	45.5	Ref	
**Number of children**						
None	65	76.5	20	23.5	2.56	0.01
One	74	59.2	51	40.8	1.14	0.675
Two	74	46	87	54	0.67	0.191
Three	62	47.7	68	52.3	0.72	0.295
Four	40	44.9	49	55.1	0.64	0.191
Five	36	45.6	43	54.4	0.66	0.229
Six and above	33	55.9	26	44.1	Ref	

COR: crude odds ratio; CI: confidence interval

The study sought to find out if education, age, and occupation had an association with unmet needs for family planning. The findings indicate that women with low levels of education were more susceptible to myths, which led to low contraceptives uptake. The younger women also encountered stigma on the utilization of contraceptives, lacked youth-tailored support, and thus increased their unmet needs. The findings from the inferential analysis and interviews point out to a direction that there exists an association between the social-demographic characteristics and the unmet family planning needs.

**Association of knowledge on family planning with unmet need for family planning:** an analysis of the association between knowledge about family planning and unmet need for family planning is summarized in Table 5. Women who never received information on family planning were significantly higher (COR = 7.73; 95%CI = 2.70 - 22.08; p < 0.001) compared to those women who ever received information. Similarly, women who were aware of any family planning methods had significantly more unmet need compared to those women who were not aware (p <0.001 using Fisher´s Exact Test). Additionally, women who lacked awareness of the benefits of contraceptive use had significantly higher unmet need for family planning (COR = 19.84; 95%CI = 2.66 - 148.32; p = 0.004).

**Table 5:** log odds on association between knowledge of family planning and unmet need for family planning

Variables	Unmet need		Met need		COR	p value
	n	%	n	%		
**Ever received any information on family planning?**						
No	32	88.9	4	11.1	7.73	<0.001
Yes	352	50.9	340	49.1	Ref	
**Awareness of any family planning methods**						
No	19	100	0	0	UD	
Yes	365	51.5	344	48.5	Ref	
**Awareness of the benefits of using contraceptives in family planning**						
No	21	95.5	1	4.5	19.84	0.004
Yes	363	51.4	343	48.6	Ref	
None/slightly/moderate	146	59.1	101	40.9	1.48	0.014
Very/extremely	238	49.5	243	50.5	Ref	
**Overall level of knowledge**						
Low/moderate (<80%)	158	60.3	104	39.7	1.61	0.002
High (80% and above)	226	48.5	240	51.5	Ref	

Women with limited confidence in understanding of contraceptives had a significantly more unmet need for family planning (COR = 1.48; 95%CI = 1.08 - 2.01; p = 0.014) compared to those women who were very or extremely confident. The proportion of unmet need was significantly more (COR = 1.61; 95%CI = 1.19 - 2.19; p = 0.002) among women with a low or moderate level of knowledge on family planning compared to those women with a high level of knowledge.

The findings showed that while there is basic knowledge on contraceptives, there was limited understanding of how the contraceptives work, the side effects as well as the associated benefits with their use. There are still misconceptions that contraceptives cause infertility and grave harm to the bodies, such as serious illnesses, which still fuels fears of their usage. Some WRA also lack knowledge on the range of available methods, and where to access the services. The challenges have been worsened by the illiteracy levels, limited access to health education. Both inferential and the thematic analysis point out of an existence of an association between the knowledge and the unmet family planning needs.

**Relationship between attitude towards family planning and unmet need for family planning:** a bivariate analysis of the relationship between attitude towards family planning and unmet need for family planning is shown in Table 6. Women who perceived family planning for controlling family size as not important or somewhat important had a significantly higher likelihood of unmet need (COR = 1.74; 95%CI = 1.16 - 2.60; p = 0.007) compared to those who perceived it very important. Women who disagreed or were neutral about the benefits for well-being and health had significantly higher unmet need (COR = 1.96; 95%CI = 1.20 - 3.21; p = 0.008) compared to those who agreed with its benefits. Likewise, the proportion of unmet need was significantly higher (COR = 1.47; 95%CI = 1.06 - 2.04; p = 0.021) among women who disagreed or remained neutral about society´s support for family planning being judgmental rather than supportive. Women who were not confident or remained neutral about accessing and use of family planning had a significantly more unmet need for family planning, with a COR of 2.21 (95%CI = 1.40 - 3.50; p = 0.001). The proportion of women who disagreed or were neutral about raising awareness of family planning in the community was significantly higher to have likelihood of unmet need (COR of 2.38; 95%CI = 1.17 - 4.86; p = 0.017). Women with a low or moderate level of attitude towards family planning had a significantly higher unmet need for family planning (COR = 1.53; 95%CI = 1.09 - 2.16; p = 0.015) compared to those women with a high level of attitude.

**Table 6:** log odds on the relationship between attitude towards family planning and unmet need for family planning

Variables	Unmet need		Met need		COR	p value
	n	%	n	%		
**Family planning is important in controlling the size of families**						
Not /somehow important	78	63.9	44	36.1	1.74	0.007
Very important	306	50.5	300	49.5	Ref	
**Family planning is meant to benefit the well-being and health of women**						
Disagree / neutral	53	67.1	26	32.9	1.96	0.008
Agree	331	51	318	49	Ref	
**Access to a variety of family planning methods is important in enabling women to make decisions that are important for their reproductive health**						
Disagree / neutral	44	62.9	26	37.1	1.58	0.077
Agree	340	51.7	318	48.3	Ref	
**An open discussion between partners is crucial in fostering healthy relationships with spouses**						
Disagree / neutral	59	62.1	36	37.9	1.55	0.051
Agree	325	51.3	308	48.7	Ref	
**Support by society on family planning is more judgmental than supportive**						
Disagree / neutral	121	59.6	82	40.4	1.47	0.021
Agree	42	51.9	39	48.1	Ref	
**Quite confident in accessing and use of family planning when needed**						
Disagree / neutral	67	69.1	30	30.9	2.21	0.001
Agree	317	50.2	314	49.8	Ref	
**Family planning needs to be promoted in the community**						
Disagree / neutral	28	71.8	11	28.2	2.38	0.017
Agree	356	51.7	333	48.3	Ref	

COR: crude odds ratio; CI: confidence interval

Health practitioners asked whether there were specific cultural and social attitudes that influence family planning use among women of reproductive age in Siaya County noted that many women still face pressure from partners or family members who believe that using contraceptives is against cultural or religious norms. Myths still exist such as infertility, loss of libido, or health complications from contraceptive use. Besides, most of the communities still belief having many children is an economic security and social status. The factors have lowered the uptake of contraceptives due to fears of stigmatization.

## Discussion

According to the study findings, 28.6% of the women wanted to avoid pregnancy for at least two years, but they were not using family planning methods. Further analysis of sociodemographic variables showed that women between 15 and 24 years had higher chances for unmet needs of family planning compared to those between 35-49 years. This study's findings corroborate a similar study in Ethiopia, which found that older age amongst women of reproductive age lowered the risk of unmet need for family planning [18]. Additionally, women without formal education had more chances of unmet family planning needs than those with formal education. Single women had a greater likelihood of unmet family planning needs than those who were married, highlighting the crucial role that socio-demographic characteristics play in the determination of family planning needs. This study's findings corroborate a study in western Kenya on improving newborn health and maternal outcomes through community-based education, where socio-demographic qualities played a significant role in enhancing the child and maternal outcomes [8].

Unmet family planning needs were significantly higher in women who never received any information on family planning than in those who received it, highlighting the importance of giving information on family planning to reduce unmet family planning needs. Besides, those who did not know the benefits of family planning had significantly higher levels of unmet family planning needs than those who were aware, which points to the need for providing information and knowledge on the benefits of using contraceptives. This finding corroborates a similar study in Kenya, which identified that adolescent and young women in Kenya with limited knowledge about contraceptive methods were significantly more likely to experience unmet need for family planning. The study emphasized the importance of increasing awareness and education on family planning to reduce unmet needs among this population [19].

Participants who perceived the importance of family planning in controlling the size of families as not important had a higher likelihood of unmet family needs than those who perceived it as very important. Besides, those who disagreed that family planning is meant to benefit the well-being and health of women in reproductive age had more chances for unmet needs than those who agreed that family planning is crucial and offers immense benefits for the well-being and health of women. The study also found that women who disagreed that open discussion with their partners is crucial in fostering a healthy relationship with their spouses were more likely to have unmet family planning needs. Participants who did not believe in the need for the promotion of family needs within the community were more likely to have unmet family needs than those who agreed on the need for the promotion of needs for family planning in the community. A study in Ghana on the expansion of health care coverage to address unmet family planning needs points in the same direction, underscoring the importance of shaping attitudes through education to boost uptake of contraceptives and reduce unmet family planning needs [2].

Unsupportive health facility officers increased the likelihood of unmet family planning needs. Women who had encountered challenges obtaining certain contraceptives from a health facility were more likely to experience unmet family planning needs. Participants who were dissatisfied with the family planning health services received from nearby health facilities were more likely to face unmet family planning needs. The findings agree with a study that found that women accessing family planning services from lower-tier health facilities, such as health centers and dispensaries, had higher odds of unmet need compared to those who accessed services from hospitals. This association was attributed to challenges like stockouts, inadequate staffing, and limited-service options in lower-level facilities, leading to dissatisfaction and reduced contraceptive uptake among women [20]. Additionally, facilities where contraceptives were not always available were more likely to contribute to unmet family planning needs than those where the contraceptives were always available, highlighting the significant contribution of health facility factors in the determination of unmet family planning needs.

## Conclusion

This study underscores the multifaceted nature of unmet family planning needs among women of reproductive age in Siaya County. Social demographic factors, in particular marital status, age, level of education, and employment status, were associated with unmet family planning needs, indicating that younger, single women without formal jobs and education were particularly vulnerable. Additionally, knowledge and information levels came up as critical determinants, with information inadequacy on contraceptive use, lack of awareness, and low confidence in understanding contraceptive use methods coming out strongly as contributors to high unmet needs for family planning. Negative perceptions of the benefits of family planning, limited spousal communication, and low community support further aggravated the unmet family planning needs. Collectively, this study advocates for an integrated approach that enhances information dissemination, strengthens health services delivery, improves attitudes to effectively reduce unmet family planning needs, and improves reproductive health outcomes among women in Siaya County.

**Recommendations:** the study recommends for an age specific training in family planning to address unique challenges faced by women in different age groups this is as a result of the findings that younger WRA who were between 15-24 years had higher unmet needs for family planning due to stigmatization, and limited access to youth friendly services. Tailored interventions that include youth-focused family planning clinics and peer education using instructional materials that enhance understanding, acceptance, and ultimately uptake of the contraceptives. Besides, it would be crucial to have training programs for older women, which should address the issues of child spacing, limiting births, considering their age, and health status. The study recommends a robust communication strategy that is aimed at closing the information gaps, more so for women who have never received any information on family planning. The approach should entail leveraging interpersonal communication through community health workers, use of digital platforms, and mass media, which should address the language barrier while at the same time being culturally sensitive, especially for the rural women, marginalized and uneducated WRA. Efforts should be made to ensure that the information given is evidence-based and accurate to correct the misconceptions about family planning methods. The study recommends for a behavioral change communication programs to address the myths and misconceptions about family planning with the goal of bringing about a positive perception of family planning through emphasizing the social, economic, and health benefits.

**Limitations:** the geographical vastness of Siaya County posed a logistical challenge to the investigator and the data collectors, who spent extra time and resources to ensure the participants were reached. The topic of family planning being an emotive subject called for the intervention of the community health promoters to ensure participation. Lastly, data security concerns and confidentiality of the participants who needed assurance that their private information would not appear in the final report.

### What is known about this topic

- The study is aware of the high unmet family planning needs amongst women of reproductive age in Siaya County, against the national average for unmet family planning needs;
- The statistics are already documented by the Ministry of Health, showing worrying concerns for a desire to have immediate action;
- Furthermore, there is evidence showing myths and misconceptions concerning the use of family planning, with many having negative myths about the use of family planning.

### What this study adds

- This study provides a baseline assessment of the unmet family planning needs levels in Siaya County;
- The study justifies the need to carry out a community-based health education intervention and assess the family planning unmet need levels at the end line of the survey after intervention;
- The study also highlights the challenges in the provision of family planning, such as information deficiency and negative attitudes towards contraceptives.
